# Tetra­methyl­ammonium (*Z*)-*N*′-cyano­carbamimidate

**DOI:** 10.1107/S2414314621010981

**Published:** 2021-11-04

**Authors:** Ray J. Butcher, Andrew P. Purdy

**Affiliations:** a Department of Chemistry, Howard University, 525 College Street NW, Washington DC 20059, USA; b Chemistry Division, Code 6123, Naval Research Laboratory, 4555 Overlook Av, SW, Washington DC 20375-5342, USA; Goethe-Universität Frankfurt, Germany

**Keywords:** crystal structure, cyano­urea salt, tetra­methyl­ammonium salt

## Abstract

In the structure of the tetra­methyl ammonium salt of cyano­urea, C_4_H_12_N^+^·C_2_H_2_N_3_O^−^, the N—C and O—C bond distances in the cyano and keto groups are in the normal range for such a moieties at 1.1641 (18) and 1.2550 (16) Å. However, the bonds about the central C and N atoms are much shorter than would be expected for single bonds and indicate that there is considerable electron delocalization in the anion as was also found in the silver salt. The NH_2_ group is coplanar with the central N_2_CO core, in contrast with the nitrile group where the dihedral angle between the N—C—N and N_2_CO planes is 36.5 (3)°. The packing of the cations and anions in the unit cell involves N—H⋯O hydrogen bonds between anions characterized by an *R* ^2^_2_(8) motif, as well as N—H⋯O hydrogen bonds between anions and C—H⋯O inter­actions between both cations and anions, forming an *R* ^3^_3_(14) pattern.

## Abstract 2

The structure of the tetra­methyl­ammonium salt of cyano­urea is reported.

## Graphical abstract

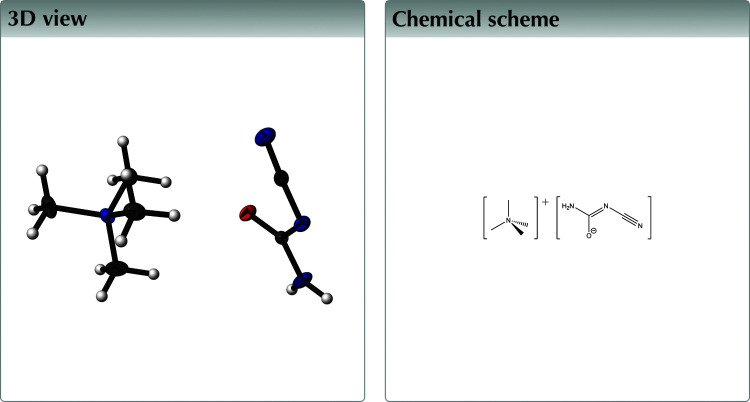

## Structure description

Cyano­urea and its salts have been the subject of much inter­est including the use of its derivatives in the study of solid state reaction mechanisms (Lotsch & Schnick, 2004 ▸), as substituents in manipulating the conformation of calix[4]arenes (Ling *et al.*, 2014 ▸), in the synthesis of amide-acid chloride adducts in organic synthesis (Harris, 1981 ▸), and in modulating the magnetic properties of Mn_6_ clusters (Yang *et al.*, 2009 ▸). In spite of this inter­est there has been very little structural characterization of this moiety and only structures of its ammonium (Lotsch & Schnick, 2004 ▸), silver (Britton, 1987 ▸), and potassium salts (Magomedova & Zvonkova, 1974 ▸) have been reported.

**Figure 1:**
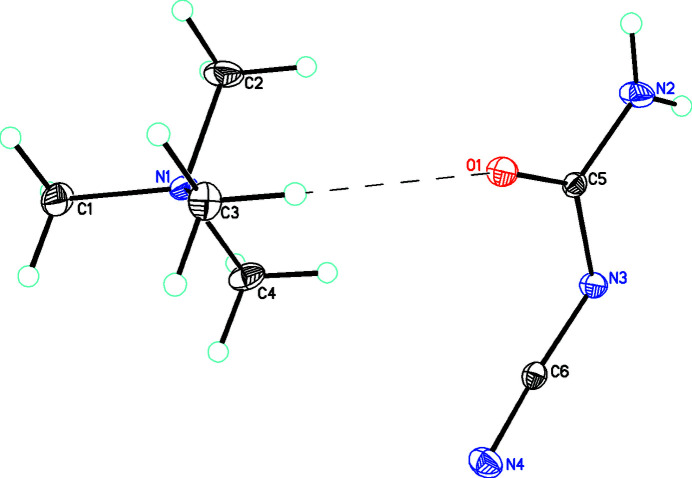
Diagram showing the [C_4_H_12_N]^+^ cation and [C_2_H_2_N_3_O_3_]^−^ anion linked by a C—H⋯O inter­action (shown as a dashed line). Atomic displacement parameters are drawn at the 30% probability level.

In the title compound, [C_4_H_12_N]^+^[C_2_H_2_N_3_O]^−^, **1**, the tetra­methyl ammonium salt of cyano­urea is reported and shown in Fig. 1 ▸. The N—C and O—C bond distances in the cyano and keto groups [1.1641 (18) and 1.2550 (16) Å,respective] are in the normal range for such a moieties and similar to the values found for the silver salt [1.149 (6) and 1.248 (5) Å, respectively]. However, the bonds about C5 and N3 are much shorter than would be expected for single bonds (Table 1 ▸) and indicate that there is considerable electron delocalization in the anion, as was also found in the silver salt. In **1**, the NH_2_ group is coplanar with the central N_2_CO core [dihedral angle between NH_2_ and N_2_CO planes of only 0.54 (8)°] in contrast with the nitrile group where the dihedral angle between the N—C—N and N_2_CO planes is 36.5 (3)°. These values are different to those found in the silver salt where the corresponding angles are 23 (6) and 4.5 (3)°.

**Figure 2:**
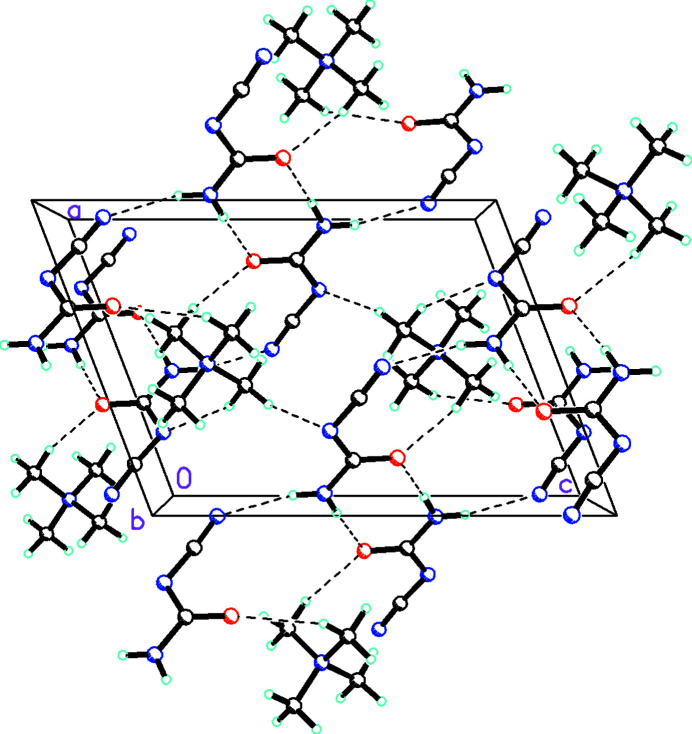
Diagram showing the packing of the cations and anions in the unit cell, which involves N—H⋯O hydrogen bonds between anions characterized by an 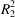 (8) motif as well as N—H⋯O hydrogen bonds between anions and C—H⋯O inter­actions between both cations and anions forming an 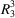 (14) pattern (all inter­actions shown with dashed lines).

The packing of the cations and anions in the unit cell involves N—H⋯O hydrogen bonds (Table 2 ▸) between anions characterized by an 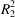 (8) motif as well as N—H⋯O hydrogen bonds between anions and C—H⋯O inter­actions between both cations and anions forming an 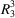 (14) pattern as shown in Fig. 2 ▸.

## Synthesis and crystallization

An ion-exchange column packed with Dowex HCR-W2 resin was regenerated with 3*M* HCl and washed with water. A solution of 5.00 g of NaN(CN)_2_ was run through the column and the product was neutralized with Me_4_NOH until alkaline. The solution was roto-vapped to dryness, recrystallized from EtOH, washed with MeOH and recrystallized from EtOH again, and pumped to dryness to afford about 1 g of product. Apparently the dicyanamide was partially hydrolyzed to form cyano­urea when in free acid form.

NMR of Me_4_N^+^ H_2_NC(O)NCN^−^ (D_2_O) ^1^H: δ3.06; ^13^C (DSS ref): δ58.0 (Me_4_N, ^1^J_C—N_ = 4 Hz), 127.0(C≡N), 171.1 (C=O); ^15^N(NH_4_NO_3_ ref): δ22.5 (Me_4_N), 62.22 (*m*, NH_2_), 72.18 (N), 150.45 (C≡N).

## Refinement

Crystal data, data collection and structure refinement details are summarized in Table 3 ▸. The structure was refined as a two-component twin with a fractional contribution of 0.0409 (11) for the minor domain.

## Supplementary Material

Crystal structure: contains datablock(s) I. DOI: 10.1107/S2414314621010981/bt4118sup1.cif

Structure factors: contains datablock(s) I. DOI: 10.1107/S2414314621010981/bt4118Isup2.hkl

Supporting information file. DOI: 10.1107/S2414314621010981/bt4118Isup3.cml

CCDC reference: 2116890

Additional supporting information:  crystallographic information; 3D view; checkCIF report

## Figures and Tables

**Table 1:** Selected geometric parameters (Å, °)

O1—C5	1.2550 (16)	N3—C5	1.3703 (17)
N2—C5	1.3464 (18)	N4—C6	1.1641 (18)
N3—C6	1.3155 (19)		
			
C6—N3—C5	114.79 (12)	N2—C5—N3	114.95 (12)
O1—C5—N2	120.31 (12)	N4—C6—N3	174.73 (15)
O1—C5—N3	124.73 (13)		

**Table 2:** Hydrogen-bond geometry (Å, °)

*D*—H⋯*A*	*D*—H	H⋯*A*	*D*⋯*A*	*D*—H⋯*A*
C1—H1*C*⋯O1^i^	0.98	2.63	3.487 (2)	147
C2—H2*A*⋯O1^i^	0.98	2.57	3.447 (2)	149
C3—H3*B*⋯O1^i^	0.98	2.62	3.484 (2)	147
C3—H3*C*⋯O1	0.98	2.30	3.253 (2)	164
C4—H4*A*⋯N3^ii^	0.98	2.54	3.450 (2)	155
C4—H4*C*⋯N3^iii^	0.98	2.59	3.536 (2)	162
N2—H2*D*⋯O1^iv^	0.88	2.03	2.9084 (16)	174
N2—H2*E*⋯N4^v^	0.88	2.18	3.0126 (19)	158

Symmetry codes: (i) 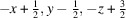 ; (ii) 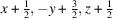 ; (iii) 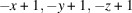 ; (iv) 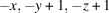 ; (v) 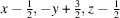 .

**Table 3:** Experimental details

Crystal data	
Chemical formula	C_4_H_12_N^+^·C_2_H_2_N_3_O^−^
*M*_r_	158.21
Crystal system, space group	Monoclinic, *P*2_1_/*n*
Temperature (K)	100
*a*, *b*, *c* (Å)	8.8120 (4), 8.7561 (4), 12.1093 (6)
β (°)	110.897 (2)
*V* (Å^3^)	872.88 (7)
*Z*	4
Radiation type	Mo *K*α
μ (mm^−1^)	0.09
Crystal size (mm)	0.25 × 0.12 × 0.05

Data collection	
Diffractometer	Bruker APEXII CCD
Absorption correction	Multi-scan (*SADABS*; Bruker, 2016 ▸)
*T*_min_, *T*_max_	0.651, 0.747
No. of measured, independent and observed [*I* > 2σ(*I*)] reflections	17548, 4340, 2752
*R*_int_	0.156
(sin θ/λ)_max_ (Å^−1^)	0.836

Refinement	
*R*[*F* ^2^ > 2σ(*F* ^2^)], *wR*(*F* ^2^), *S*	0.081, 0.174, 1.04
No. of reflections	4340
No. of parameters	105
H-atom treatment	H-atom parameters constrained
Δρ_max_, Δρ_min_ (e Å^−3^)	0.44, −0.27

Computer programs: *APEX2* and *SAINT* (Bruker, 2016 ▸), *SHELXT* (Sheldrick 2015*a* ▸), *SHELXL2018/3* (Sheldrick, 2015*b* ▸), and *SHELXTL* (Sheldrick 2008 ▸).

## full crystallographic data

### Crystal data

**Table:**

C_4_H_12_N^+^·C_2_H_2_N_3_O^−^	*F*(000) = 344
*M**_r_* = 158.21	*D*_x_ = 1.204 Mg m^−^^3^
Monoclinic, *P*2_1_/*n*	Mo *K*α radiation, λ = 0.71073 Å
*a* = 8.8120 (4) Å	Cell parameters from 3088 reflections
*b* = 8.7561 (4) Å	θ = 2.9–36.0°
*c* = 12.1093 (6) Å	µ = 0.09 mm^−^^1^
β = 110.897 (2)°	*T* = 100 K
*V* = 872.88 (7) Å^3^	Prism, colourless
*Z* = 4	0.25 × 0.12 × 0.05 mm

### Data collection

**Table:**

Bruker APEXII CCD diffractometer	2752 reflections with *I* > 2σ(*I*)
φ and ω scans	*R*_int_ = 0.156
Absorption correction: multi-scan (Sadabs; Bruker, 2016)	θ_max_ = 36.4°, θ_min_ = 2.9°
*T*_min_ = 0.651, *T*_max_ = 0.747	*h* = −14→14
17548 measured reflections	*k* = −14→14
4340 independent reflections	*l* = −20→20

### Refinement

**Table:**

Refinement on *F*^2^	0 restraints
Least-squares matrix: full	Hydrogen site location: inferred from neighbouring sites
*R*[*F*^2^ > 2σ(*F*^2^)] = 0.081	H-atom parameters constrained
*wR*(*F*^2^) = 0.174	*w* = 1/[σ^2^(*F*_o_^2^) + (0.064*P*)^2^ + 0.1366*P*] where *P* = (*F*_o_^2^ + 2*F*_c_^2^)/3
*S* = 1.04	(Δ/σ)_max_ < 0.001
4340 reflections	Δρ_max_ = 0.44 e Å^−^^3^
105 parameters	Δρ_min_ = −0.27 e Å^−^^3^

### Special details

**Table:**

Geometry. All esds (except the esd in the dihedral angle between two l.s. planes) are estimated using the full covariance matrix. The cell esds are taken into account individually in the estimation of esds in distances, angles and torsion angles; correlations between esds in cell parameters are only used when they are defined by crystal symmetry. An approximate (isotropic) treatment of cell esds is used for estimating esds involving l.s. planes.
Refinement. Refined as a 2-component twin. The structure was solved using *SHELXT* (Sheldrick, 2015*a*) and refined with *SHELXL2018* (Sheldrick, 2015*b*). The locations of all hydrogen atoms for the major component were located in difference Fourier maps and refined in idealized position using a riding model with atomic displacement parameters of *U*_iso_(H) = 1.2*U*_eq_(C, N) [1.5*U*_eq_(C) for CH_3_], with N—H distance of 0.88 Å and C—H distances ranging from 0.95 to 0.99 Å, respectively.

### Fractional atomic coordinates and isotropic or equivalent isotropic displacement parameters (Å^2^)

**Table:**

	*x*	*y*	*z*	*U*_iso_*/*U*_eq_	
N1	0.52001 (13)	0.39529 (14)	0.77065 (11)	0.0155 (2)	
C1	0.65132 (18)	0.3158 (2)	0.86761 (18)	0.0271 (3)	
H1A	0.717795	0.391510	0.923711	0.041*	
H1B	0.719731	0.257919	0.834033	0.041*	
H1C	0.602538	0.245780	0.908689	0.041*	
C2	0.4173 (2)	0.2805 (2)	0.68548 (17)	0.0289 (3)	
H2A	0.367659	0.211613	0.726730	0.043*	
H2B	0.484911	0.221365	0.651990	0.043*	
H2C	0.331794	0.333191	0.621804	0.043*	
C3	0.41712 (17)	0.4843 (2)	0.82257 (14)	0.0215 (3)	
H3A	0.484635	0.559198	0.878912	0.032*	
H3B	0.367829	0.414567	0.863515	0.032*	
H3C	0.331387	0.537379	0.759369	0.032*	
C4	0.5940 (2)	0.5019 (2)	0.70750 (15)	0.0238 (3)	
H4A	0.666165	0.573678	0.764306	0.036*	
H4B	0.507855	0.558664	0.647290	0.036*	
H4C	0.656586	0.443175	0.669525	0.036*	
O1	0.16653 (13)	0.63130 (14)	0.57913 (9)	0.0193 (2)	
N2	0.04603 (16)	0.59992 (17)	0.38199 (11)	0.0232 (3)	
H2D	−0.024142	0.534851	0.391904	0.028*	
H2E	0.041806	0.623148	0.310245	0.028*	
N3	0.26105 (14)	0.76503 (15)	0.45058 (11)	0.0172 (2)	
N4	0.46972 (16)	0.89392 (18)	0.61890 (12)	0.0230 (3)	
C5	0.15986 (15)	0.66395 (15)	0.47658 (12)	0.0139 (2)	
C6	0.36986 (15)	0.82986 (16)	0.54310 (12)	0.0150 (2)	

### Atomic displacement parameters (Å^2^)

**Table:**

	*U*^11^	*U*^22^	*U*^33^	*U*^12^	*U*^13^	*U*^23^
N1	0.0151 (4)	0.0119 (4)	0.0204 (5)	−0.0002 (4)	0.0074 (4)	0.0002 (4)
C1	0.0182 (6)	0.0206 (7)	0.0386 (9)	0.0056 (5)	0.0053 (6)	0.0070 (7)
C2	0.0337 (7)	0.0235 (7)	0.0285 (8)	−0.0118 (6)	0.0099 (7)	−0.0097 (7)
C3	0.0202 (5)	0.0247 (7)	0.0223 (6)	0.0058 (5)	0.0109 (5)	0.0006 (5)
C4	0.0322 (7)	0.0196 (7)	0.0257 (7)	−0.0061 (5)	0.0179 (6)	−0.0015 (5)
O1	0.0229 (4)	0.0226 (5)	0.0125 (4)	−0.0056 (4)	0.0062 (4)	0.0014 (4)
N2	0.0280 (5)	0.0266 (6)	0.0124 (5)	−0.0125 (5)	0.0040 (4)	−0.0013 (5)
N3	0.0199 (5)	0.0182 (5)	0.0130 (4)	−0.0040 (4)	0.0052 (4)	0.0008 (4)
N4	0.0232 (5)	0.0280 (7)	0.0167 (5)	−0.0061 (5)	0.0058 (4)	−0.0017 (5)
C5	0.0156 (5)	0.0121 (5)	0.0137 (5)	0.0006 (4)	0.0047 (4)	0.0002 (4)
C6	0.0160 (5)	0.0155 (5)	0.0148 (5)	0.0013 (4)	0.0069 (4)	0.0022 (4)

### Geometric parameters (Å, º)

**Table:**

N1—C2	1.492 (2)	C3—H3C	0.9800
N1—C3	1.4937 (17)	C4—H4A	0.9800
N1—C1	1.494 (2)	C4—H4B	0.9800
N1—C4	1.4958 (19)	C4—H4C	0.9800
C1—H1A	0.9800	O1—C5	1.2550 (16)
C1—H1B	0.9800	N2—C5	1.3464 (18)
C1—H1C	0.9800	N2—H2D	0.8800
C2—H2A	0.9800	N2—H2E	0.8800
C2—H2B	0.9800	N3—C6	1.3155 (19)
C2—H2C	0.9800	N3—C5	1.3703 (17)
C3—H3A	0.9800	N4—C6	1.1641 (18)
C3—H3B	0.9800		
			
C2—N1—C3	109.42 (12)	N1—C3—H3B	109.5
C2—N1—C1	109.76 (14)	H3A—C3—H3B	109.5
C3—N1—C1	109.17 (12)	N1—C3—H3C	109.5
C2—N1—C4	109.55 (13)	H3A—C3—H3C	109.5
C3—N1—C4	109.31 (12)	H3B—C3—H3C	109.5
C1—N1—C4	109.62 (12)	N1—C4—H4A	109.5
N1—C1—H1A	109.5	N1—C4—H4B	109.5
N1—C1—H1B	109.5	H4A—C4—H4B	109.5
H1A—C1—H1B	109.5	N1—C4—H4C	109.5
N1—C1—H1C	109.5	H4A—C4—H4C	109.5
H1A—C1—H1C	109.5	H4B—C4—H4C	109.5
H1B—C1—H1C	109.5	C5—N2—H2D	120.0
N1—C2—H2A	109.5	C5—N2—H2E	120.0
N1—C2—H2B	109.5	H2D—N2—H2E	120.0
H2A—C2—H2B	109.5	C6—N3—C5	114.79 (12)
N1—C2—H2C	109.5	O1—C5—N2	120.31 (12)
H2A—C2—H2C	109.5	O1—C5—N3	124.73 (13)
H2B—C2—H2C	109.5	N2—C5—N3	114.95 (12)
N1—C3—H3A	109.5	N4—C6—N3	174.73 (15)
			
C6—N3—C5—O1	−0.5 (2)	C6—N3—C5—N2	178.60 (13)

### Hydrogen-bond geometry (Å, º)

**Table:**

*D*—H···*A*	*D*—H	H···*A*	*D*···*A*	*D*—H···*A*
C1—H1*C*···O1^i^	0.98	2.63	3.487 (2)	147
C2—H2*A*···O1^i^	0.98	2.57	3.447 (2)	149
C3—H3*B*···O1^i^	0.98	2.62	3.484 (2)	147
C3—H3*C*···O1	0.98	2.30	3.253 (2)	164
C4—H4*A*···N3^ii^	0.98	2.54	3.450 (2)	155
C4—H4*C*···N3^iii^	0.98	2.59	3.536 (2)	162
N2—H2*D*···O1^iv^	0.88	2.03	2.9084 (16)	174
N2—H2*E*···N4^v^	0.88	2.18	3.0126 (19)	158

Symmetry codes: (i) −*x*+1/2, *y*−1/2, −*z*+3/2; (ii) *x*+1/2, −*y*+3/2, *z*+1/2; (iii) −*x*+1, −*y*+1, −*z*+1; (iv) −*x*, −*y*+1, −*z*+1; (v) *x*−1/2, −*y*+3/2, *z*−1/2.
